# Maternal employment status and child age are positive determinants of minimum dietary diversity among children aged 6–23 months in Sagnarigu municipality, Ghana: a cross-sectional study

**DOI:** 10.1186/s40795-024-00865-7

**Published:** 2024-04-15

**Authors:** Ambrose Atosona, Jawahir Abukari Mohammed, Huzaifa Issahaku, Khadija Saani, Hammond Yaw Addae, Fusta Azupogo

**Affiliations:** 1 Department of Nutritional Sciences, School of Allied Health Sciences, University for Development Studies, Tamale, Ghana; 2 Nursing and Midwifery Training College, Kpembe, Ghana; 3 Faculty of Agriculture, Department of Family and Consumer Sciences, Food and Consumer Sciences, University for Development Studies, Tamale, Ghana

**Keywords:** Minimum dietary diversity, Nutrient adequacy, Children, Ghana

## Abstract

**Background:**

Intake of a diversified diet is key to the prevention of malnutrition among children as it results in improved intake of energy and micronutrients, which are deemed critical for better nutritional status of children. This study assessed minimum dietary diversity (MDD) and its determinants among children aged 6–23 months in the Sagnarigu Municipality of Ghana.

**Methods:**

This was an analytical cross-sectional study, carried out in the Sagnarigu Municipality, Ghana and involved 369 mother-child pairs selected through a systematic random sampling. A semi-structured questionnaire was used to obtain respondents’ socio-demographic characteristics, feeding practices, nutritional knowledge and anthropometry. MDD was assessed using a repeated 24-hour dietary recall method. Chi-square/Fisher exact test and binary logistic regression analysis were used to determine the factors associated with MDD.

**Results:**

The study found that 24.9% of the children were between the ages of 6–8 months and 75.1% were between the ages of 9–23 months. About 64.2% of the children met the MDD. Children of mothers who were self-employed were approximately 2 times more likely to meet the MDD as compared to children of mothers who were unemployed [Adjusted Odd Ratio (AOR): 1.93, 95% CI (1.13–3.31), *P* = 0.017]. Also, children aged 9–23 months were approximately 14 times more likely to meet MDD as compared to younger children aged 6–8 months [AOR: 13.98, 95% CI (7.54–25.91), *P* < 0.001].

**Conclusion:**

Our study suggests that maternal empowerment may have positive effects on improving the MDD of infants and young children.

## Background

Childhood malnutrition, particularly chronic malnutrition (stunting) remains the most problematic public health problem globally [1]. Stunting affects 22% [2] of the global population, 28.6% [3] of children in Sub-Saharan Africa and 18% of children in Ghana [4]. Ghana’s Northern and North-East Regions have significantly higher rates of stunting, affecting approximately 30% of infants and young children [4]. Stunting prevalence stands at 47.6% [5] in the Sagnarigu Municipality.

Malnutrition in children results in delays in physical and motor development, reduced intelligence, more behavioural problems, poor social skills, and increased susceptibility to diseases [6, 7]. Almost 45% of mortalities in children under the age of five are caused by undernutrition and these largely occur in nations with low and intermediate levels of income [1]. More than two-thirds of these undernutrition-related mortalities are linked to inappropriate feeding practices in the first two years of life [8]. The rapid growth during this period increases the children’s nutrient requirements, making them more susceptible to malnutrition [9]. Hence, appropriate feeding practices during this period (appropriate, safe, adequate and frequent feeding) are critical for their optimal growth and development [10].

MDD, a diet quality indicator for children aged 6–23 months [11], is defined as the intake of at least five or more food groups from the recommended eight food groups: (1) Breast milk, (2) grains, roots and tubers, (3) legumes and nuts, (4) dairy products, (5) flesh foods, (6) eggs, (7) vitamin A rich fruits and vegetables and (8) other fruits and vegetables [12]. It is one of the several indicators developed by World Health Organization (WHO) to offer simple, accurate, and reliable metrics for evaluating infant and young child feeding practices (IYCF) at the population level [13].

Meeting the MDD is key to the prevention of malnutrition among children as it results in improved intake of energy and nutrients which are deemed critical for better nutritional status [14]. Yet, many parents, particularly those in the low-and middle-income countries struggle to provide diversified diets for their children [3]. Globally, less than a quarter of children aged 6 to 23 months meet the MDD score and feeding frequency [15]. In Sub-Saharan Africa and Ghana, only 25.1% [3] and 35.3% of children aged 6–23 months receive MDD [16] respectively. The lack of variety in children’s diets in most Sub-Sahara Africa nations has been attributed to household food insecurity [17, 18]. Maternal education, nutritional knowledge, number of children, antenatal and postnatal care visits and household monthly income have been identified as key determinants of MDD among children aged 6 to 23 months in some settings [19–22]. Identifying context-specific factors influencing MDD is pivotal in designing effective interventions. This tailored approach holds promise in mitigating childhood malnutrition, as it enables strategies that account for diverse local conditions and individual needs. By addressing these determinants, we can enhance the effectiveness of efforts aimed at improving children’s nutrition and overall well-being. Despite MDD being a known major determinant of child nutritional status [14] and the fact that the prevalence rate of child malnutrition (stunting) is extremely high (47.6%) in the Sagnarigu Municipality of Ghana [5], MDD and its context specific determinants in the Sagnarigu Municipality are currently unknown. This study, therefore, aimed to assess MDD and its determinants among children aged 6–23 months in the Sagnarigu Municipality of Ghana.

## Methods

### Study design, area and population

An analytical cross-sectional study design was used in the present study. The study was conducted in Kalpohini, Kpalsi, Sagnarigu and Wurshe communities in the Sagnarigu municipality of Northern Ghana from August to October, 2022. Eligible participants were mother-child pairs with the children aged 6–23 months who consented to participate in the study. Participants who were not sound minded were excluded from the study.

### Sampling

Multi-stage sampling method was used in this study. Four study communities were selected through simple random sampling. All the 79 communities under Sagnarigu Municipality were listed and assigned specific numbers written on pieces of paper, put in a basin, mixed, and picked one at a time with replacement till the number of communities were reached. Systematic random sampling was used to select the households, where the households were selected according to a random starting point but with a fixed interval. This interval was calculated by dividing the total number of households by the desired sample size. In households with two or more eligible children, only one child was selected through simple random sampling. The population proportion to size method was utilized to calculate the sample size for each of the communities.

### Sample size

The sample size for this study was calculated using the Cochran’s formula:

N$$ =\frac{{z}^{2}p(1-p)}{{M.E}^{2}}$$ [23]

Where:

N is the sample size.

z is confidence interval (95%) which gives a critical value of 1.96.

p is the estimated proportion of an attribute present in the population. Prevalence of minimum dietary diversity is 35.3% in northern Ghana [16].

M.E is the desired level of precision (5%=0.05).

Hence, *N* = 351. A 5% contingency was considered to cover up incomplete questionnaires. Hence, *N* = 369.

### Study variables

#### Dependent variable

The dependent variable was MDD.

#### Independent variables

The independent variables were caregiver characteristics (age, marital status, ethnicity, religion, educational level, occupation, income level, birth interval, antenatal care visits, post-natal care visits, place of delivery, breastfeeding status, timely initiation of breastfeeding and nutritional knowledge) and child characteristics (age, sex and history of illness in the past 2 weeks).

### Data collection

#### Pretesting of questionnaire

The questionnaire pretesting was done by administering the questionnaires to 37 (10% of total sample size) caregivers with children aged 6–23 months in the study district; these caregivers did not participate in the study. This allowed for the researchers to fine-tune the questions for clarity and comprehension by the participants.

#### Socio-demographic characteristics

The pretested semi-structured questionnaire was used to take information on socio-demographic characteristics of the mother including age, marital status, ethnicity, religion, educational level, occupation, income level and sex of child. Data collection was facilitated by two trained research assistants.

#### Health service utilization and obstetric characteristics

Participants’ characteristics including antenatal care (ANC) visits, post-natal care (PNC) visits, place of delivery and birth interval were also documented.

#### Caregivers knowledge on dietary diversity and feeding practices

Caregivers’ knowledge on dietary diversity and feeding practices was assessed using a questionnaire adapted from Solomon et al. [24]. The questionnaire consisted of ten knowledge questions. Each correct answer (yes) was assigned a score of 1, while any wrong answer was assigned a zero (0) score. Mothers who got a score of 7 and above out of the ten knowledge questions were deemed to have good knowledge while mothers who got a score of less than 7 were deemed to have poor knowledge [24].

#### Dietary diversity

Data on MDD was collected using the WHO indicators for assessing IYCF practices [25]. A 24-hour dietary recall, repeated in 20% of random sub-sample [26], was used to obtain food intake information. The 24-hour dietary recall was conducted on two non-consecutive days, one weekday and one weekend [27–29]. The respondents were asked to recall all foods eaten and beverages taken by the children in the previous 24 h prior to the interview. The dietary diversity score (DDS) was assessed by assigning a score of 1 to a food group if the child ate any food item from the food group and a score of 0 if no food item from the food group was consumed. A total of 8 food groups [25] were considered in this study: Breast milk; grains, roots and tubers; legumes, nuts and seeds; dairy products; flesh foods, eggs; vitamin A-rich fruits and vegetables and other fruits and vegetables. Consequently, the minimum possible DDS score was 0 (no food group consumed) and the maximum possible DDS score was 8 (all food groups consumed). A child was classified as having achieved the MDD if he/she consumed at least 5 food groups out of the 8.

#### Anthropometry

Length (cm) of child was measured without footwear using an infantometer (Seca, Germany) and weight (kg) was measured without clothing using a digital weighing scale (Seca, Germany). The child’s age, sex, and measurements of weight and length were used to calculate the following growth indicators: length-for-age (stunting), weight-for-age (underweight) and weight-for-length (wasting) in accordance with the WHO 2006 child growth reference [30]. The cut off point for stunting, wasting and underweight was − 2SD from the median of WHO child growth standard.

### Data analysis

The Statistical Package for Social Science (SPSS version 22) was used to analyze the data. The results were reported with descriptive statistics including frequencies, percentages, means, and standard deviations. Chi-square/Fisher exact test was used for bivariate analysis. The factors with *p* < 0.25 [31] in the bivariate analysis were selected for multivariate logistic regression analysis to determine predictors of MDD. *P* < 0.05 (at two-tailed test) was considered significant.

## Results

### Socio-demographic characteristics

The study had a 100% response rate. Close to two-thirds of the mothers were within 20–30 years of age (62.6%). The predominant religion was Islam (95.1%), and nearly all mothers were married (99.5%). Also, the main ethnic group was Dagombas (90.2%), with the majority (89.1%) having a household monthly income of less than 500 Ghana cedis. Also, the majority (66.4%) of the mothers had less than 5 children. Regarding education, most of the mothers (46.1%) did not have any formal education. The majority of the children were males (51.8%) and aged 9–23 months (75.1%) (Table 1).

**Table 1:** Socio-demographic characteristics

Variable	Category	Frequency	Percentage
Mother’s age (in years)	< 20	21	5.7
	20–30	231	62.6
	31–40	111	30.1
	> 40	6	1.6
Religion	Christianity	18	4.9
	Islam	351	95.1
Maternal marital status	Single	1	0.3
	Married	367	99.5
	Separated	1	0.3
Number of children	< 5	247	66.4
	6–10	97	26.3
	≥ 11	25	6.8
Household monthly income	< Ghc500	329	89.1
	Ghc 500–999	25	6.8
	Ghc 1000–1500	10	2.7
	> Ghc1500	5	1.4
Ethnicity	Dagomba	333	90.2
	Gonja	12	3.3
	Others	24	6.5
Employment status	Self-employed	203	55.0
	Employed	23	6.2
	Unemployed	143	38.8
Mother’s highest educational level completed	None	170	46.1
	Primary	65	17.6
	Middle/JHS	58	15.7
	SHS/vocational training	40	10.8
	Tertiary	36	9.8
Age of child (in months)	6–8	92	24.9
	9–23	277	75.1
Sex of child	Male	191	51.8
	Female	178	48.2

### Feeding practices, maternal nutritional knowledge and nutritional status of children

**Fig. 1:**
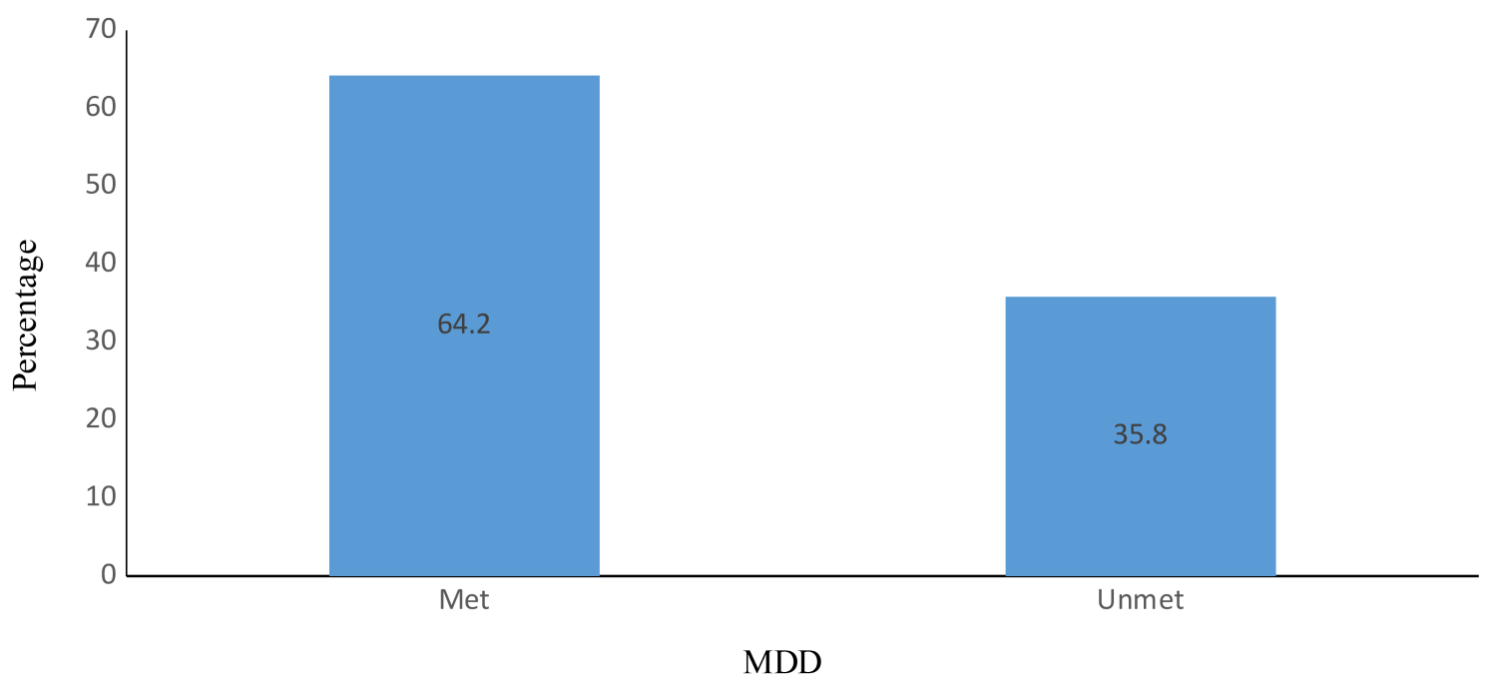
The percentage of children who met the minimum dietary diversity (MDD)

In the present study, about 96.5% of the children were breastfed. About two-thirds (66.7%) of them started breastfeeding within the first hour of delivery. With regards to mother’s knowledge on IYCF practices, the majority (77.0%) had good knowledge. About 32.8% of the children fell ill 2 weeks prior to the study. The prevalence of wasting, stunting and underweight were 20.3%, 24.4% and 24.4% respectively (Table 2). Of the total number of children, 237 (64.2%) met the MDD score (Fig. 1).

**Table 2:** Feeding practices, maternal nutritional knowledge and nutritional status of children

Variable	Category	Frequency	Percentage
Currently breastfeeding	Yes	356	96.5
	No	13	3.5
Timely initiation of breastfeeding	Yes	246	66.7
	No	123	33.3
Nutritional knowledge of mother	Good	285	77
	Poor	86	23
Stunted	Yes	90	24.4
	No	279	75.6
Wasted	Yes	75	20.3
	No	294	79.7
Underweight	Yes	90	24.4
	No	279	75.6
Child history of illness in the past 2 weeks	Yes	121	32.8
	No	248	67.2

### Health service utilization and obstetric characteristics

About two-thirds (66.9%) of the mothers delivered at the hospital. Almost all (99.5%) mothers attended antenatal care services, with the majority (92.1%) of them going for more than 4 visits. The number of mothers who attended postnatal care visits was 363 (98.4%). Also, about two-thirds of the mothers (64.5%) had a birth interval of more than 2 years (Table 3).

**Table 3:** Health service utilization and obstetric characteristics

Variable	Category	Frequency	Percentage
Place of delivery	At home	90	24.4
	Health center	32	8.7
	Hospital	247	66.9
Antenatal care visits	Yes	367	99.5
	No	2	0.5
Frequency of antenatal care visits	1	4	1.1
	2–3	23	6.2
	≥ 4	340	92.1
	None	2	0.5
Post-natal care visits	Yes	363	98.4
	No	6	1.6
Birth interval	1 year	91	24.6
	2 years	40	10.8
	More than 2 years	238	64.5

### Factors associated with minimum dietary diversity practice

Significant determinants of MDD at the bivariate level included employment status (*p* = 0.119), educational level (*p* = 0.153), frequency of antenatal care visits (*p* = 0.027), sex of child (*p* = 0.059) and age of child (*p* < 0.001) (Table 4).

**Table 4:** Bivariate analysis of factors associated with minimum dietary diversity

Characteristic	Total population *N* = 369(%)	MDD UNMET= 35.8(%)	MDD MET= 64.2(%)	P-Value
**Age of mother**				
<20 20–30	21(100) 231(100)	5(23.8) 90(39.0)	16(76.2) 141(61.0)	0.325
31–40	111(100)	36(32.4)	75(67.6)	
>40	6(100)	1(16.7)	5(83.3)	
**Ethnicity**				
Dagomba	333(100)	118(35.4)	215(64.6)	
Gonja	12(100)	5(41.7)	7(58.3)	0.883
Others	24(100)	9(37.5)	15(62.5)	
**Religion**				
Christianity	18(100)	6(33.3)	12(66.7)	
Islam	351(100)	126(35.9)	225(64.1)	0.825
**Marital status**				
Single	1(100)	0(0.0)	1(100.0)	
Married	367(100)	132(36.0)	235(64.0)	1.000
Separated	1(100)	0(0.0)	1(100.0)	
**Employment status**				
Self-employed	203(100)	66(32.5)	137(67.5)	
Employed	23(100)	6(26.1)	17(73.9)	0.119
Unemployed	143(100)	60(42.0)	83(58.0)	
**Education**				
None	170(100)	64(37.6)	106(62.4)	
Primary	65(100)	19(29.2)	46(70.8)	
Middle/JHS	58(100)	16(27.6)	42(72.4)	0.153
SHS/vocational training	40(100)	20(50.0)	20(50.0)	
Tertiary	36(100)	13(36.1)	23(63.9)	
**Household income**				
< GH¢ 500	329(100)	113(34.3)	216(65.7)	
GH¢ 500–999	25(100)	15(60.0)	10(40.0)	0.27
GH¢ 1000–1500	10(100)	2(20.0)	8(80.0)	
> GH¢ 1500	5(100)	2(40.0)	3(60.0)	
**Period between birth**				
1 year	91(100)	40(43.9)	51(56.1)	0.260
2 years	40(100)	13(32.5)	27(67.5)	
> 2	238(100)	79(33.2)	159(66.8)	
**ANC attendance**				
Yes	367(100)	131(35.7)	236(64.3)	
No	2(100)	1(50.0)	1(50.0)	1.000
**Frequency of ANC visits**				
Once	4(100)	3(75.0)	1(25.0)	
2–3 times	23(100)	13(56.5)	1(25.0)	0.027
≥4 times	341(100)	116(34.0)	225(66.0)	
**PNC attendance**				
Yes	363(100)	130(35.8)	233(64.2)	
No	6(100)	2(33.3)	4(66.7)	1.000
**Sex of child**				
Male	191(100)	77(40.3)	114(59.7)	
Female	178(100)	55(30.9)	123(69.1)	0.059
**Age of child**				
6–8	92(100)	72(78.3)	20(21.7)	
9–23	277(100)	60(21.7)	217(78.3)	< 0.001
**Illness in the past 2 weeks**				
Yes	121(100)	39(32.2)	82(67.8)	
No	248(100)	93(37.5)	155(62.5)	0.322
**Knowledge of mother**				
Poor knowledge	85(100)	32(37.6)	53(62.4)	
Good knowledge	284(100)	100(35.2)	184(64.8)	0.681
**Early initiation of breastfeeding**				
Met	247(100)	86(34.8)	161(65.2)	
Unmet	121(100)	45(37.2)	76(62.8)	0.655

In the multivariable binary logistic regression analysis, employment status and age of child were the predictor variables. Children of mothers who were self-employed were twice more likely to meet the MDD as compared to children of mothers who were unemployed [AOR: 1.93, 95% CI (1.13–3.31), *p* = 0.017]. The children aged 9–23 months were about 14 times more likely to meet MDD as compared to younger children (6–8 months) [AOR: 13.98, 95% CI (7.54–25.91), *p* < 0.001] (Table 5).

**Table 5:** Multivariable analysis of factors associated with minimum dietary diversity

Characteristics	Unadjusted odds ratio (95% confidence interval)	P- value	Adjusted odds ratio (95% confidence interval)	P- value
**Age (months)**				
6–8	1		1	
9–23	13.02(7.35–23.07)	< 0.001	13.98 (7.54–25.91)	< 0.001
**Employment status**				
Unemployed	1	1		
Self-Employed	0.67(0.43–1.04)	0.073	1.930 (1.13–3.31)	0.017
Employed	0.49 (0.182–1.31)	0.155	3.496 (0.834–14.652)	0.087
**Education**				
None	1		1	
Primary	0.68 (0.369–1.27)	0.229	1.541 (0.74–3.20)	0.247
Middle/JHS	0.63 (0.328–1.21)	0.168	2.14 (0.97–4.71)	0.059
SHS/vocational	1.66 (0.0828 − 3.31)	0.154	0.854 (0.36–2.014)	0.719
Tertiary	0.94(0.443–1.98)	0.863	0.648 (0.21–1.98)	0.446
**ANC attendance**				
Once	1		1	
2–3 times	0.43 (0.04–4.82)	0.496	3.38 (0.22–50.92)	0.379
≥4 times	0.17 (0.02–1.67)	0.129	3.64 (0.03–45.27)	0.315
**Sex of child**				
Female	1		1	
Male	1.51(0.98–2.32)	0.060	1.07(0.64–1.80)	0.786

## Discussion

The consumption of a well-diversified diet prevents childhood malnutrition as it improves the intake of nutrients and energy. This study investigated the prevalence of MDD and its predictors among children aged 6–23 months in the Sagnarigu Municipality of Ghana.

The study revealed that about 64.2% of the children met the MDD score which was higher than the national average of 41% reported in the 2022 Ghana Demographic and Health Survey Report [4] This is higher than the rate reported in a cross-sectional study in Ghana (35.3%) [16]. Similarly, the rate reported in the current study is also higher than the rates reported in Ethiopia [32] and Bangladesh [33], where the rates were found to be 12.6% and 28.7% respectively. On the other hand, the finding of the present study is comparable to that of Solomon et al. [24] and Sekartaji et al. [34], who reported rates of 59.6% and 63.2% respectively. This variation in results may partly be attributed to differences in methodologies [35, 36], population characteristics [35], nutrition knowledge of mothers [37], economic conditions [38], women`s empowerment and food security [39].

The present study also revealed a general trend towards higher dietary diversity as children age. The children aged 9–23 months were approximately 14 times more likely to meet the MDD as compared to younger children (6–8 months). This finding is consistent with the findings of several studies [32, 40–42]. It could be that children typically acquire a broader range of dietary preferences as they grow older, leading to increased diversity in their family meal choices. This might be a result of late introduction of complementary foods and when introduced on time, only milk or cereal foods like porridge are usually given, as some mothers have the perception that, the younger the child, the poorer the ability of the child to digest other foods such as beef, eggs, green leafy vegetables, carrots, and bananas [32, 43]. Also, teething and increased infections during infancy (6–8 months) lead to loss of appetite, resulting in low dietary intake and low dietary diversity [38]. This may explain the finding of the present and previous studies [43, 44]. Our finding emphasises the need for tailored nutritional interventions to address the specific needs of different age groups within the early childhood period. This could involve nutrition education on transitioning from exclusive breastfeeding to the introduction of solid foods and ensuring a diverse diet.

In the present study, children of mothers who were self-employed (traders, farmers, hairdresser etc.) were twice more likely to meet the MDD as compared to children of mothers who were unemployed. In conformity with the finding of the present study, studies by Issaka et al. [45] and Belay et al. [43] revealed that working mothers are more likely to feed their children diversified diets as compared to non-working (unemployed) mothers. This might be the case because mothers who work are more likely to have higher incomes which may translate to higher purchasing power, thereby improving household food security [46]. Increased household food security is associated with increased MDD among children in Ghana [47]. The stark difference in MDD achievement between children of self-employed mothers and those of unemployed mothers underscores the potential impact of economic stability and parental employment on child nutrition. Policies aimed at promoting and supporting self-employment opportunities, or income generation activities for women could have a beneficial impact on child nutrition.

Furthermore, child’s sex, maternal educational status, and number of ANC visits by mothers were not significantly correlated with MDD at the multivariate level in the present study. In line with this finding, a similar study by Amoah et al. [48] revealed no relationship between MDD and these three factors. Contrarily, Sema et al. [49], in a study that determined the MDD and related factors among children aged 6–23 months, failed to show a correlation between MDD and child’s gender, maternal educational status and number of ANC visits by mothers. The mixed findings could, at least in part, be attributed to the methodological variances among the studies [35, 36].

This study is not without limitations. The cross-sectional nature of the study makes it difficult to establish causal associations as exposure and outcome were measured simultaneously. Also, being a recall and self-reported study, recall bias might have affected the exact estimation of minimum dietary diversity. Despite these, the study has shed insight into MDD and related characteristics among children aged 6 to 23 months in Sagnarigu Municipality for the first time. It is recommended that longitudinal studies should be carried out to establish cause and effect relationship between the dependent and independent variables.

## Conclusion

The level of MDD practice was high in the study area. Maternal employment status and age of child were the significant determinants of MDD. Mothers, especially those unemployed, should be encouraged to feed their children diversified diets with emphasis on the transition period from exclusive breastfeeding to complementary feeding so as to reduce the risks of malnutrition.

## Data Availability

The datasets used and/or analysed during the current study are available from the corresponding author on reasonable request.

## Abbreviations

MDD: Minimum dietary diversity
WHO: World Health Organization
ANC: Antenatal care
PNC: Post-natal care
IYCF: Infant and young child feeding
CM: Centimeters
KG: Kilogram
SPSS: Statistical Package for Social Sciences
AOR: Adjusted Odds Ratio
